# Essential Oils as a Dietary Additive for Small Ruminants: A Meta-Analysis on Performance, Rumen Parameters, Serum Metabolites, and Product Quality

**DOI:** 10.3390/vetsci9090475

**Published:** 2022-09-02

**Authors:** Griselda Dorantes-Iturbide, José Felipe Orzuna-Orzuna, Alejandro Lara-Bueno, Germán David Mendoza-Martínez, Luis Alberto Miranda-Romero, Héctor Aarón Lee-Rangel

**Affiliations:** 1 Departamento de Zootecnia, Universidad Autónoma Chapingo, Texcoco 56230, Mexico; 2 Departamento de Producción Agrícola y Animal, Unidad Xochimilco, Universidad Autónoma Metropolitana, Mexico City 04960, Mexico; 3 Centro de Biociencias, Universidad Autónoma de San Luis Potosí, San Luis Potosí 78321, Mexico

**Keywords:** carcass traits, enteric methane, antioxidant status, meat quality, milk quality

## Abstract

**Simple Summary:**

Essential oils can be used to improve animal performance as well as the health and quality of livestock products. The aim of this study was to evaluate the effects of essential oil supplementation on animal performance, ruminal fermentation, blood metabolites, and meat and milk quality of small ruminants through a meta-analysis. Supplementation with essential oils improved weight gain, milk production and composition, oxidative stability of meat, and blood serum antioxidant enzyme activity. Additionally, essential oils decreased methane emissions. This suggests that the inclusion of essential oils in the diets of small ruminants could be used to improve animal performance and the quality of meat and milk, in addition to reducing the environmental impact and oxidative stress of the animals.

**Abstract:**

There is an increasing pressure to identify natural feed additives that improve the productivity and health of livestock, without affecting the quality of derived products. The objective of this study was to evaluate the effects of dietary supplementation with essential oils (EOs) on productive performance, rumen parameters, serum metabolites, and quality of products (meat and milk) derived from small ruminants by means of a meta-analysis. Seventy-four peer-reviewed publications were included in the data set. Weighted mean differences (WMD) between the EOs treatments and the control treatment were used to assess the magnitude of effect. Dietary inclusion of EOs increased (*p* < 0.05) dry matter intake (WMD = 0.021 kg/d), dry matter digestibility (WMD = 14.11 g/kg of DM), daily weight gain (WMD = 0.008 kg/d), and feed conversion ratio (WMD = −0.111). The inclusion of EOs in small ruminants’ diets decreased (*p* < 0.05) ruminal ammonia nitrogen concentration (WMD = −0.310 mg/dL), total protozoa (WMD = −1.426 × 10^5^/mL), methanogens (WMD = −0.60 × 10^7^/mL), and enteric methane emissions (WMD = −3.93 L/d) and increased ruminal propionate concentration (WMD = 0.726 mol/100 mol, *p* < 0.001). The serum urea concentration was lower (WMD = −0.688 mg/dL; *p* = 0.009), but serum catalase (WMD = 0.204 ng/mL), superoxide dismutase (WMD = 0.037 ng/mL), and total antioxidant capacity (WMD = 0.749 U/mL) were higher (*p* < 0.05) in response to EOs supplementation. In meat, EOs supplementation decreased (*p* < 0.05) the cooking loss (WMD = −0.617 g/100 g), malondialdehyde content (WMD = −0.029 mg/kg of meat), yellowness (WMD = −0.316), and total viable bacterial count (WMD = −0.780 CFU/g of meat). There was higher (*p* < 0.05) milk production (WMD = 0.113 kg/d), feed efficiency (WMD = 0.039 kg/kg), protein (WMD = 0.059 g/100 g), and lactose content in the milk (WMD = 0.100 g/100 g), as well as lower somatic cell counts in milk (WMD = −0.910 × 10^3^ cells/mL) in response to EOs supplementation. In conclusion, dietary supplementation with EOs improves productive performance as well as meat and milk quality of small ruminants. In addition, EOs improve antioxidant status in blood serum and rumen fermentation and decrease environmental impact.

## 1. Introduction

In ruminants, antibiotics have been used for several years to prevent and cure diseases, as well as to improve growth and the efficiency of conversion of ingested feed into products for human consumption, such as meat and milk [1]. However, due to the inappropriate use of antibiotics, the emergence of bacteria with resistance to their effects is currently among the main threats to global health [2]. In addition, the extensive use of antibiotics in ruminants can generate antibiotic residues in meat and milk, which when consumed by humans can affect their health [3]. Therefore, in recent years, the interest in the use of natural products to improve the health and productivity of livestock has increased [4]. Among these, EOs are plant-derived products that have gained greater economic relevance [5]. EOs extracted from plants are obtained by distillation and are composed of mixtures of low-weight molecules, such as terpenes (monoterpenes and sesquiterpenes), terpenoids, ketones, aldehydes, and alcohols [6].

It has been reported that EOs and their bioactive metabolites have diverse biological effects, such as antimicrobial, anti-inflammatory, antioxidant, and antiparasitic, among others [6,7]. In previous studies, the effects of EOs as dietary additives have been evaluated mainly in non-ruminants [8,9,10]. Therefore, information on the effects of dietary inclusion of EOs in ruminants is still limited. However, there is evidence that EOs can improve the efficiency of energy utilization and nutrient intake by ruminants [11]. Moreover, moderate doses of some EOs in a diet can improve volatile fatty acid production and protein metabolism in ruminants [12], while, at high doses, some EOs can decrease methane production [13]. In contrast, EOs have been reported to have no positive effects on productive performance and nutrient utilization efficiency in ruminants [1]. Furthermore, a large part of the positive effects of EOs on ruminal fermentation have been obtained from in vitro studies and using high doses [11], which, when applied to in vivo studies in ruminants, are likely to negatively affect feed intake [14] and ruminal fermentation [11].

Particularly in small ruminants, some studies have evaluated the effect of dietary inclusion of EOs on animal performance [15,16], nutrient digestibility, ruminal fermentation [17,18], blood biochemistry [19,20], and meat quality [21,22], as well as milk production and composition [23,24]. However, no conclusive results have been obtained until now, perhaps due to the variability that exists among these studies regarding the experimental periods, primary bioactive compounds, and the doses of EOs used [25]. Thus, identifying and controlling this variability could help to obtain EOs that can be used in small ruminants to improve animal performance, rumen fermentation, health, and product quality.

Several review articles have been published to date [1,5,12,13,14] concluding that dietary supplementation with EOs can be used to improve animal productive performance, rumen parameters, animal health, and product quality (meat and milk) in ruminants. These positive effects of EOs have been confirmed in beef cattle and dairy cows using meta-analytical methods [25,26]. In a previous meta-analysis (MA), Khiaosa-ard and Zebeli [27] evaluated the effects of dietary inclusion of EOs on rumen fermentation in small ruminants. However, that study [27] only included six references applied to small ruminants in their database and did not evaluate productive performance, blood metabolites, or meat and milk quality. Although the meta-analytical approach has been used mainly in research related to human health, its application in research on natural food additives in domestic animals is still limited [28,29]. MA allows to combine and quantitatively synthesize previously published results from multiple independent studies [30]. In addition, with the use of MA, it is possible to identify sources of heterogeneity among studies performed on the same subject [31]. 

Considering the mentioned antecedents, the hypothesis of the present study proposes that the addition of EOs in diets for small ruminants will benefit productive performance, rumen parameters, and meat and milk quality without affecting animal health. For this reason, the objective of this meta-analysis was to evaluate the effects of dietary supplementation with essential oils on productive performance, carcass characteristics, nutrient digestibility, ruminal parameters, serum metabolites, and meat and milk quality of small ruminants.

## 2. Materials and Methods

### 2.1. Literature Search and Study Selection

**Figure A1:**
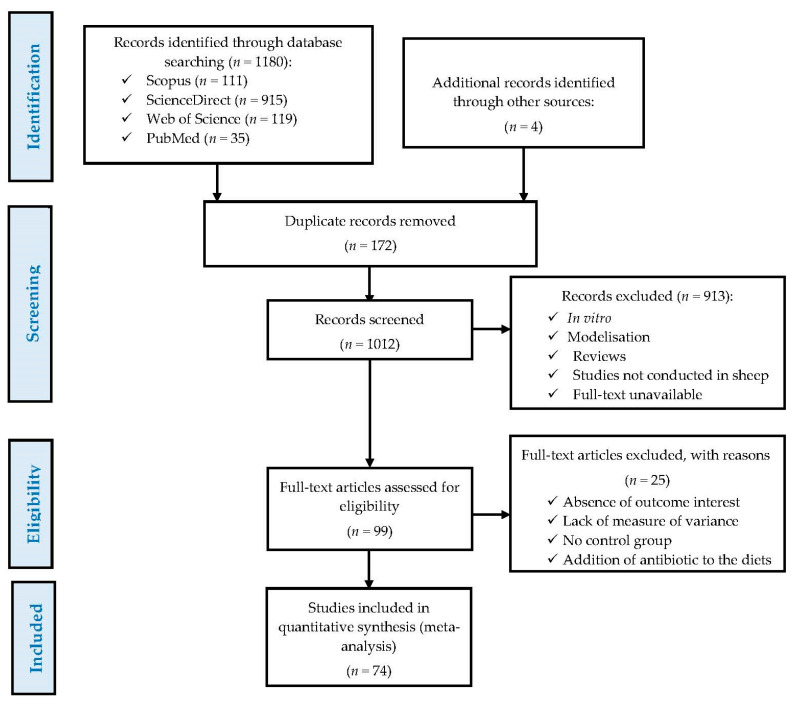
A PRISMA flow diagram detailing the literature search strategy and study selection for the meta-analysis.

For this meta-analysis, PRISMA guidelines [32] were followed during the identification, selection, and inclusion of previous studies, as shown in Figure A1. To identify previous studies that evaluated the effects of dietary inclusion of EOs on animal performance, carcass characteristics, nutrient digestibility, ruminal parameters, serum metabolites, as well as meat and milk quality of small ruminants, a systematic search for information was performed in the PubMed, Web of Science, ScienceDirect, and Scopus databases. The keywords used were essential oils, finishing lamb, growing lamb, finishing goat, growing goat, lactating goat, lactating sheep, digestibility, carcass, ruminal fermentation, blood metabolites, milk production, and meat quality. The search results were restricted to studies published between January 2010 and May 2022. In Appendix A, Figure A1 shows the 1184 scientific publications identified. When a publication was reported in more than one database, the duplicate was excluded. Subsequently, a two-step publication selection process was applied [25,30,31]. First, the titles and abstracts of each publication were reviewed to exclude studies that were not conducted in small ruminants, studies that did not measure any of the variables of interest, studies that used infected small ruminants, reviews, simulation articles, and in vitro experiments.

Second, to be included in the final database, the articles analyzed had to meet some inclusion criteria, similar to those previously reported by Orzuna-Orzuna et al. [25,30,31]: (1) studies that used small ruminants (sheep and goats) housed under confinement conditions; (2) data on nutrient digestibility, animal performance, carcass characteristics, serum metabolites, ruminal parameters, and milk quality and/or meat quality are available; (3) studies that had control and experimental treatments with similar feeding, except for the presence of EOs in the diets; (4) studies that indicated the dose of EOs used or have sufficient information to estimate the dose of EOs included in the diets; (5) studies that were written in English and published in peer-reviewed scientific journals; and (6) studies that reported the means of the control and experimental treatments with standard deviation or standard error and the number of replicates.

### 2.2. Data Extraction

Based on the inclusion criteria, only 74 articles were included in the database for the final analysis (Table A1). Only response variables that were reported in at least three studies were included in the database [25,30,31]. Therefore, among the variables included in the present meta-analysis were the following: dry matter intake, dry matter and nutrient digestibility (protein and ethereal extract, among others), daily weight gain, feed conversion ratio, carcass characteristics (hot carcass weight and yield, among others), ruminal parameters (volatile fatty acids, protozoa and bacteria, and pH and ammonia nitrogen), serum metabolites (urea, glucose and cholesterol, among others), antioxidant enzymes in blood serum (superoxide dismutase and catalase, among others), characteristics related to meat quality (color, chemical composition, pH, and malondialdehyde content, among others), as well as milk production and composition (protein and fat and lactose content).

Finally, when available, from the 74 selected publications, the publication reference (author and year), the country where the study was conducted, the amount of forage and concentrate in the diet (g/kg DM), the nutritional composition of the diet (g/kg DM), the period of supplementation with EOs (days), the dose of EOs in the diet (mg/kg DM), and the primary bioactive metabolite of the Eos were obtained. From these publications, the number of replicates means and standard deviations (SD) for each of the control and experimental treatments were extracted. In articles where the SD was not reported, it was calculated using the standard errors of the means (SEM), using the equation [33]: SD = SEM × √*n*, where *n* = number of replicates.

### 2.3. Calculations and Statistical Analysis

To perform the meta-analysis, as well as for heterogeneity, publication bias, and meta-regression analyses, the metaphor package [34] in the statistical software R (version 4.1.2) was used. The effects of EOs as an additive in small ruminant diets were evaluated by weighted mean differences (WMD) between treatments with EOs (diets with EOs) and control treatments (diets without EOs). Treatment means were weighted with the inverse of the variance, following methods previously proposed by DerSimonian and Laird [35] for random effects models. WMD was used because it allows interpretation of the results in the original units of measurement [28].

On the other hand, the MEANS procedure of the statistical software SAS [36] was used to obtain descriptive statistical values of the nutritional composition of the diets. In addition, the MIXED procedure of SAS was used to evaluate the differences in the nutritional composition of the diets of the EOs treatments and the control treatments. For this, random effect studies were used, as well as the Tukey test to detect differences between treatments, as previously reported by Orzuna-Orzuna et al [25,30,31].

### 2.4. Heterogeneity and Publication Bias

In the meta-analysis, the heterogeneity of treatment effect was assessed with the chi-square (Q) test, in which, due to its relatively low power, a significance level of *p* ≤ 0.10 was used [37]. In addition, the I^2^ statistic was used to measure the percentage of variation due to heterogeneity [29]. Negative values of I^2^ (percent variation) were assigned as zero, while values less than 25, 25 to 50, and greater than 50% indicated low, moderate, and high heterogeneity, respectively [28,29].

On the other hand, publication bias was assessed using Egger’s regression asymmetry test [38], which was considered significant (publication bias) when *p* ≤ 0.05 was observed. In addition, when Egger’s test was statistically significant (*p* ≤ 0.05), the “trim and fill” method of Duval and Tweedie [39] was used with the aim of estimating the possible number of missing observations.

### 2.5. Meta-Regression and Subgroup Analysis

A meta-regression analysis was performed to identify sources of heterogeneity in the variables evaluated. The meta-regression criteria were (1) *p* ≤ 0.10 for the Q test [37] or I^2^ greater than 50% [28]; (2) *p* ≥ 0.05 for Egger’s test [38]; and (3) response variables reported in at least 10 studies [40]. In all cases, meta-regression was performed using the method of moments of DerSimonian and Laird [35], which is well-established to estimate the variance between studies. When covariates were significant, with *p* ≤ 0.05, WMD was evaluated by subgroup analysis. The primary bioactive compound (carvacrol, eugenol, thymol, limonene, and linalool, among others) was used as a categorical covariate, whereas the duration of the experimental phase (days) and the dose of EOs (mg/kg DM) were used as continuous covariates. When covariates were significant, with *p* ≤ 0.10; these were evaluated by subgroup analysis [25,30,31]. Each of the primary bioactive metabolites was considered a single category. Moreover, when meta-regression was significant (*p* ≤ 0.05) for each Eos supplementation period (days) and dietary dose of Eos (mg/kg DM), these two covariates were evaluated by the subgroups: supplementation period (≤70 and >70 days) and dietary dose of Eos (≤500, 501–1000 and >1000 mg/kg DM).

## 3. Results

### 3.1. Study Attributes and Excluded Studies

Table 1 shows that there were no differences (*p* < 0.05) between the control treatment and the different treatments with Eos for forage, concentrate, nutrients, and metabolizable energy content of the diets. This suggests that, for the data set analyzed, it is possible to exclude the effects of these components on the response of small ruminants to dietary inclusion of Eos.

In the present meta-analysis, the studies included were performed in 17 different countries, mainly in Egypt (16.2%), China (13.5%), Spain (12.2%), Iran (9.5%), Turkey (8.1%), and Tunisia (8.1%). Regarding the animal species, sheep were used in 75.7% of the studies, and goats were used in the rest (24.3%). Table A1 shows that the experimental periods ranged from 14 to 288 days, while the experimental doses of Eos ranged from 10 to 40,000 mg/kg DM. The Eos were grouped based on the primary bioactive metabolite, and in total, 20 different types of primary bioactive metabolites were observed. EOs with mixtures of primary bioactive metabolites in similar proportions were the most commonly used in the treatments (44.8%). Moreover, a significant proportion of the treatments used EOs with carnosic acid (11.6%), carvacrol (6.1%), thymol (4.9%), and limonene (4.9%) as a primary bioactive metabolite, while, in the remaining treatments (27.7%), EOs with 15 other different primary bioactive metabolites were used (Table A1).

### 3.2. Dry Matter Intake and Digestibility

Table 2 shows that dry matter intake increased (*p* < 0.001) in response to dietary supplementation of EOs. Similarly, dietary inclusion of EOs increased (*p* < 0.05) dry matter digestibility (DMD), organic matter digestibility (OMD), crude protein digestibility (CPD), neutral detergent fiber digestibility (NDFD), and acid detergent fiber digestibility (ADFD). However, ether extract (EE) digestibility was similar among treatments (*p* > 0.05).

### 3.3. Growth Performance and Carcass Characteristics

Table 3 shows that daily weight gain (DWG), hot carcass yield (HCY), and *Longissimus dorsi* muscle area (LMA) increased in response to dietary supplementation with EOs (*p* < 0.05). On the other hand, dietary inclusion of EOs decreased the feed conversion ratio (FCR; *p* = 0.045). However, hot carcass weight (HCW), cold carcass weight (CCW), and backfat thickness (BFT) were similar among treatments (*p* > 0.05).

### 3.4. Ruminal Parameters and Ruminal Microorganisms

Table 4 shows that ruminal pH was similar between treatments (*p* > 0.05). Moreover, dietary supplementation with EOs did not affect (*p* > 0.05) the rumen concentration of acetate, butyrate, and the count of *Entodinium*, *Diplodinium*, *Isotrichae*, total bacteria, *Ruminococcus albus* (*R. albus*) and *Fibrobacter succinogenes* (*F. succinogenes*). However, dietary inclusion of EOs reduced (*p* < 0.05) ruminal ammonia nitrogen (NH_3_-N) concentration, total protozoan populations, *Epidinium*, methanogens, and daily enteric methane (CH_4_) emissions. On the other hand, ruminal propionate concentration and relative amount of *Ruminococcus flavefaciens* (*R. flavefaciens*) increased in response to EOs supplementation.

### 3.5. Blood Metabolites

Table 5 shows that dietary supplementation with EOs decreased (*p* < 0.05) the serum concentration of urea, cholesterol, triglycerides, non-esterified fatty acids (NEFA), and beta-hydroxybutyrate (BHB). On the other hand, dietary supplementation with EOs did not affect (*p* > 0.05) the serum concentration of glucose, albumin, globulin, total protein, malondialdehyde (MDA), and glutathione peroxidase (GPx). However, higher serum concentrations of thyroxine, catalase (CAT), superoxide dismutase (SOD), and total antioxidant capacity (TAC) were observed in response to the dietary inclusion of EOs (*p* < 0.05).

### 3.6. Meat Quality

Dietary supplementation with EOs decreased (*p* < 0.05) cooking loss (CL), shear force (ShF), yellowness (b*), and malondialdehyde (MDA) content from day 1 to day 14 of meat storage (Table 6). Similarly, dietary inclusion of EOs decreased (*p* < 0.05) the total viable count (TVC) of bacteria, total psychrophilic bacteria (PSY), molds and yeasts (MY), and Enterobacteriaceae bacteria (ENT) in meat. On the other hand, no significant impact (*p* > 0.05) of dietary supplementation with EOs on pH, water holding capacity (WHC), lightness (L*), redness (a*), and chemical composition (protein, fat, moisture, and ash) of meat was observed (Table 6).

### 3.7. Milk Yield and Quality

Dietary supplementation with EOs increased (*p* < 0.05) milk yield, feed efficiency (FE), and protein and lactose content in milk (Table 7). On the other hand, dietary supplementation with EOs decreased somatic cell count (SCC) and milk urea content (*p* < 0.001). However, there was no significant impact (*p* > 0.05) of dietary inclusion of EOs on milk fat content and milk pH (Table 7).

### 3.8. Publication Bias and Meta-Regression 

Table 2, Table 3, Table 4, Table 5, Table 6 and Table 7 show that Egger’s asymmetry regression test was non-significant (*p* > 0.05) for all variables evaluated, which indicates that there was no publication bias.

On the other hand, Table 2, Table 3, Table 4, Table 5, Table 6 and Table 7 show significant (*p* ≤ 0.10) heterogeneity (Q) for DMD, OMD, CPD, EED, NDFD, ADFD, ADG, LMA, ruminal pH, NH_3_-N, acetate, propionate, butyrate, total protozoa, *Entodinium* protozoa, total bacteria, *R. flavefaciens*, *R. albus*, *F. succinogenes*, methanogens, glucose, cholesterol, triglycerides, NEFA, GPx, TAC, meat pH, MDA content in meat on days 3, 6 and 14 of storage, TVC, milk yield, and protein, fat, lactose, SCC, and urea content in milk. However, to obtain reliable results, it is recommended to use meta-regression only when the variable of interest is reported in at least 10 studies [40]. Consequently, meta-regression was only performed for the variables: DMD, OMD, CPD, NDFD, ADFD, ADG, LMA, rumen pH, NH_3_-N, acetate, propionate, butyrate, total protozoa, glucose, cholesterol, triglycerides, meat pH, and milk yield, as well as protein, fat, and lactose content in milk.

Table 8 shows that EOs dose explained (*p* = 0.004) 16.39% of the observed heterogeneity for ruminal NH_3_-N concentration. Similarly, EOs dose explained (*p* < 0.001) 2.65 and 28.20% of the observed heterogeneity for serum triglyceride concentration and milk yield, respectively (Table A2). On the other hand, the experimental period only explained (*p* = 0.003) 3.52 and 7.30% of the heterogeneity observed for rumen pH and total rumen protozoa (Table 8). Moreover, Table A2 shows that the experimental period explained (*p* = 0.011) 10.41% of the heterogeneity observed for milk protein content. Table 8 shows that the primary bioactive metabolite explained (*p* < 0.05) between 7.63 and 62.55% of the observed heterogeneity for DMD, NDFD, ADFD, ruminal pH, NH_3_-N, acetate, propionate, butyrate, and total protozoa. Likewise, the primary bioactive metabolite of the EOs explained (*p* < 0.05) 30.28, 80.30, 47.17, and 32.38% of the observed heterogeneity for serum glucose concentration, triglycerides, milk yield, and milk protein content, respectively (Table A2). Moreover, Table 8 shows that there was no significant relationship (*p* > 0.05) between the covariates used and the response variables ADG, OMD, and CPD. In addition, serum cholesterol concentration and milk lactose content had no significant relationship (*p* > 0.05) with any of the covariates used (Table A2).

### 3.9. Subgroup Analysis

**Figure 1:**
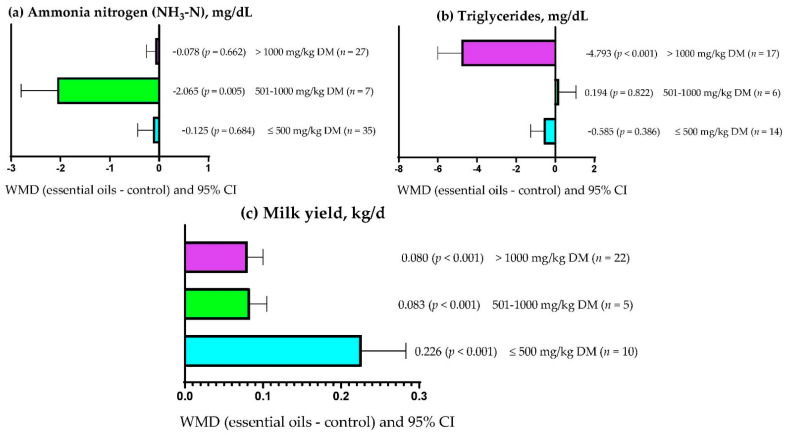
Subgroup analysis (subgroup = essential oils dose (mg/kg DM)) of the effect of essential oils on the diet of the small ruminants; WMD = weighted mean differences between essential oil treatments and control.

Figure 1a shows that ruminal NH_3_-N concentration decreased (WMD = −2.065 mg/dL; *p* = 0.005) when moderate doses of EOs (501–1000 mg/kg DM) were used. However, low (≤500 mg/kg DM) and high (>1000 mg/kg DM) doses of EOs did not affect ruminal NH_3_-N concentration. Dietary inclusion of EOs at low (501–1000 mg/kg DM) and moderate (501–1000 mg/kg DM) doses did not affect serum triglyceride concentration (Figure 1b; *p* > 0.05). However, serum triglyceride concentration decreased (WMD = −4.793 mg/dL; *p* < 0.001) when doses of EOs greater than 1000 mg/kg DM were used (Figure 1b). Figure 1c shows that milk yield increased (*p* < 0.001) regardless of the dose of EOs used. However, the effect was greater (WMD = 0.226 kg/d) when low doses of EOs (≤ 500 mg/kg DM) were used compared with doses between 501 and 1000 mg/kg DM (WMD = 0.080 kg/d) and doses greater than 1000 mg/kg DM (WMD = 0.083 kg/d).

**Figure 2:**
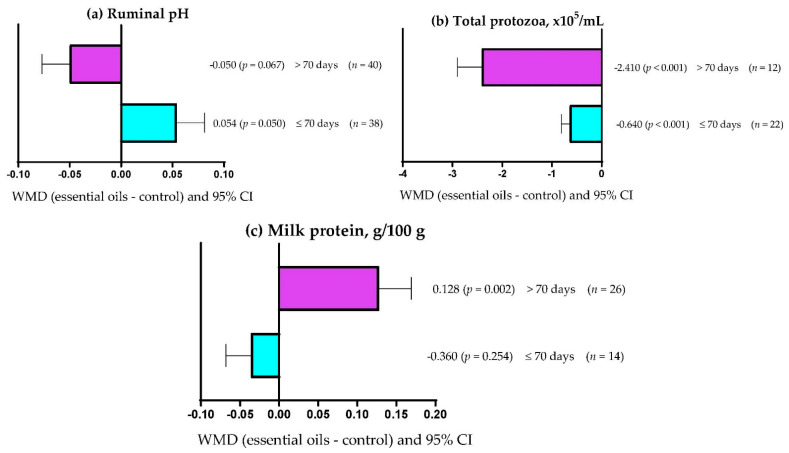
Subgroup analysis (subgroup = supplementation period (days)) of the effect of essential oils on the diet of the small ruminants; WMD = weighted mean differences between essential oil treatments and control.

Figure 2a shows that ruminal pH decreased when dietary supplementation with EOs lasted up to 70 days (WMD = 0.054; *p* = 0.050); however, pH was not affected when EOs were offered for more than 70 days (WMD = −0.054; *p* > 0.05). Rumen protozoa concentration increased (*p* < 0.001) regardless of the EO supplementation period used (Figure 2b); although, the effect was greater (WMD = −2.410 × 10^5^/mL) when EOs were offered for longer periods (>70 days) compared with periods up to 70 days (WMD = −0.640 × 10^5^/mL). On the other hand, milk protein content increased (WMD = 0.128 g/100 g; *p* = 0.002) when EOs were offered for more than 70 days (Figure 2c). However, milk protein content was not affected (WMD = −0.036 g/100 g; p = 0.254) when EOs were offered for periods up to 70 days.

**Figure 3:**
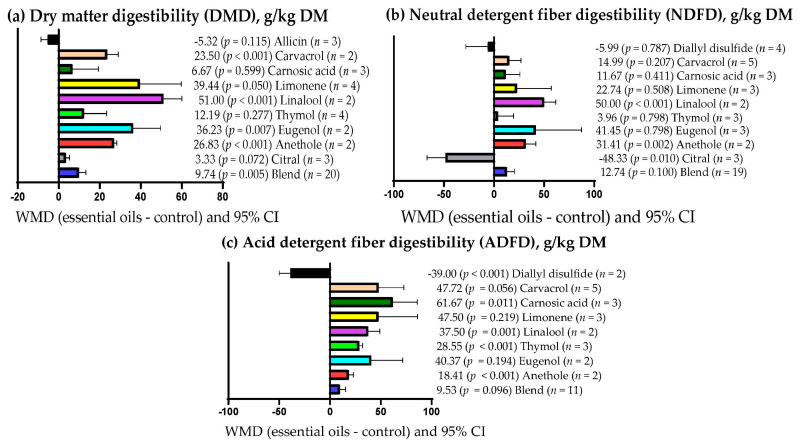
Subgroup analysis (subgroup = primary bioactive compound) of the effect of essential oils supplementation to small ruminants’ diets on their digestibility; WMD = weighted mean differences between essential oil treatments and control.

Figure 3a shows that DMD increased (*p* < 0.05) when the primary bioactive metabolites of the EOs were mixtures (WMD = 9.74 g/kg DM), carvacrol (WMD = 23.50 g/kg DM), limonene (WMD = 39.44 g/kg DM), linalool (WMD = 51.00 g/kg DM), eugenol (WMD = 36.23 g/kg DM), and anethole (WMD = 26.83 g/kg DM). However, when EOs were used with other different bioactive metabolites, DMD was not affected (*p* > 0.05). On the other hand, Figure 3b shows that NDFD increased only when the primary bioactive metabolites of the EOs were linalool (WMD = 50.00 g/kg DM; *p* < 0.001) and anethole (WMD = 31.41 g/kg DM; p = 0.002). NDFD decreased when EOs contained citral as the primary bioactive metabolite (WMD = −48.33 g/kg DM; *p* = 0.010) and was not affected when EOs with other bioactive metabolites were used (*p* > 0.05). ADFD decreased (WMD = −39.00 g/kg DM; *p* < 0.001) when EOs with diallyl disulfide as the primary bioactive metabolite were used (Figure 3c). However, ADFD increased (*p* < 0.05) when the primary bioactive metabolites of the EOs were carnosic acid (WMD = 61.67 g/kg DM), linalool (WMD = 37.50 g/kg DM), thymol (WMD = 28.55 g/kg DM), and anethole (WMD = 18.41 g/kg DM), but ADFD was not affected when EOs with other bioactive metabolites were used (*p* > 0.05).

**Figure 4:**
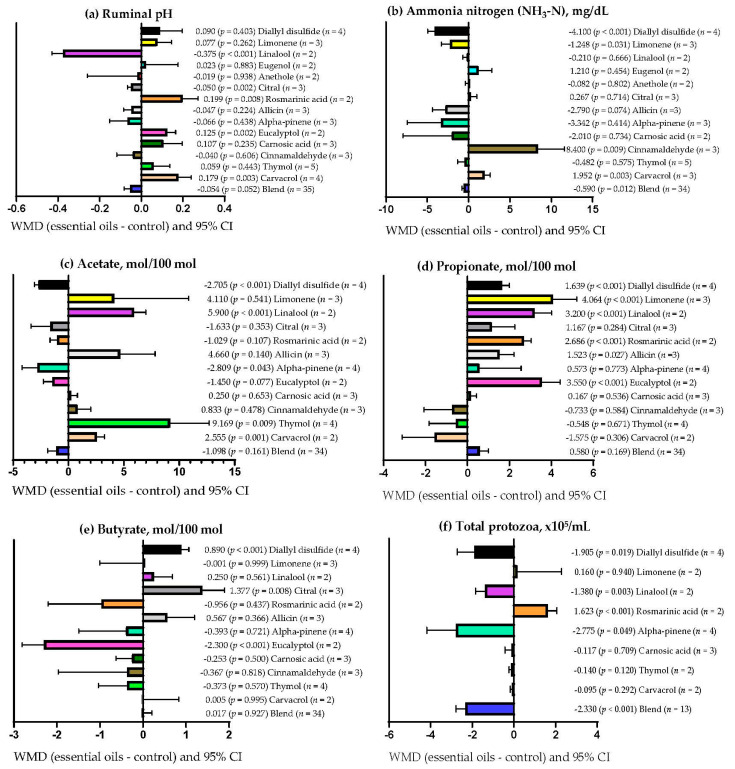
Subgroup analysis (subgroup = primary bioactive compound) of the effect of essential oils supplementation on small ruminants’ diets on their rumen parameters; WMD = weighted mean differences between essential oil treatments and control.

Figure 4a shows that ruminal pH decreased (*p* < 0.05) when the primary bioactive metabolites of the EOs were linalool (WMD = −0.375) and citral (WMD = −0.050). However, ruminal pH increased when EOs contained rosmarinic acid (WMD = 0.199), eucalyptol (WMD = 0.125), and carvacrol (WMD = 0.179) but was not affected when EOs with other bioactive metabolites were used (*p* > 0.05). Figure 4b shows that ruminal NH_3_-N concentration decreased (*p* < 0.05) only when the primary bioactive metabolites of the EOs were mixtures (WMD = −0.590 mg/dL), diallyl disulfide (WMD = −4.100 mg/dL), and limonene (WMD = −1.248 mg/dL). However, ruminal NH_3_-N concentration increased when EOs contained cinnamaldehyde (WMD = 8.400 mg/dL; *p* = 0.009) and carvacrol (WMD = 1.952 mg/dL; *p* = 0.003) as primary bioactive metabolites but was not affected when EOs with other bioactive metabolites were used (*p* > 0.05). The ruminal concentration of acetate decreased when EOs with diallyl disulfide (WMD = −2.705 mol/100 mol; *p* < 0.001) and alpha-pinene (WMD = −2.809 mol/100 mol; *p* = 0.043) were used as primary bioactive metabolites (Figure 4c). However, ruminal acetate concentration increased (*p* < 0.05) when EOs contained linalool (WMD = 5.900 mol/100 mol), thymol (WMD = 9.169 mol/100 mol), and carvacrol (WMD = 2.555 mol/100 mol) as primary bioactive metabolites but was not affected when EOs with other bioactive metabolites were used (*p* > 0.05).

The ruminal concentration of propionate increased (*p* < 0.05) when using EOs with diallyl disulfide (WMD = 1.639 mol/100 mol), limonene (WMD = 4.064 mol/100 mol), linalool (WMD = 3.200 mol/100 mol), rosmarinic acid (WMD = 2.686 mol/100 mol), allicin (WMD = 1.523 mol/100 mol), and eucalyptol (WMD = 3.550 mol/100 mol) as primary bioactive metabolites (Figure 4d). The use of EOs with other primary bioactive metabolites did not affect rumen propionate concentration (*p* > 0.05). Figure 4e shows that ruminal butyrate concentration increased only when the primary bioactive metabolites of the EOs were diallyl disulfide (WMD = 0.890 mol/100 mol; *p* < 0.001) and citral (WMD = 1.377 mol/100 mol; *p* = 0.008). Nevertheless, ruminal butyrate concentration decreased when EOs contained eucalyptol as the primary bioactive metabolite (WMD = −2.300 mol/100 mol; *p* < 0.001) and was not affected when EOs with other bioactive metabolites were used (*p* > 0.05). Additionally, Figure 4f shows that total rumen protozoa decreased (*p* < 0.05) when the primary bioactive metabolites of the EOs were mixtures (WMD = −2.330 × 10^5^/mL), diallyl disulfide (WMD = −1.905 × 10^5^/mL), linalool (WMD = −1.380 × 10^5^/mL), and alpha-pinene (WMD = −2.775 × 10^5^/mL). In contrast, total protozoa increased when EOs contained rosmarinic acid as the primary bioactive metabolite (WMD = 1.623 × 10^5^/mL; *p* < 0.001) and were not affected when EOs with other bioactive metabolites were used (*p* > 0.05).

**Figure 5:**
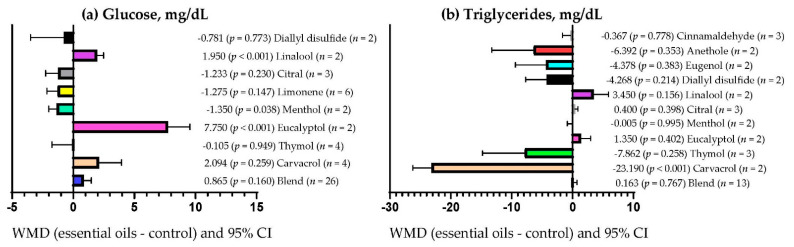
Subgroup analysis (subgroup = primary bioactive compound) of the effect of essential oils supplementation to small ruminants’ diets on their blood metabolites; WMD = weighted mean differences between essential oil treatments and control.

Figure 5a shows that serum glucose concentration increased (*p* < 0.001) only when the primary bioactive metabolites of the EOs were linalool (WMD = 1.950 mg/dL) and eucalyptol (WMD = 7.750 mg/dL), although serum glucose concentration decreased when EOs contained menthol as the primary bioactive metabolite (WMD = −1.350 mg/dL; *p* = 0.038) and was not affected when EOs with other different bioactive metabolites were used (*p* > 0.05). Serum triglyceride concentration decreased (*p* < 0.001) only when EOs contained carvacrol as the primary bioactive metabolite (WMD = −23.190 mg/dL; Figure 5b) but was not affected when EOs with other bioactive metabolites were used (*p* > 0.05). 

**Figure 6:**
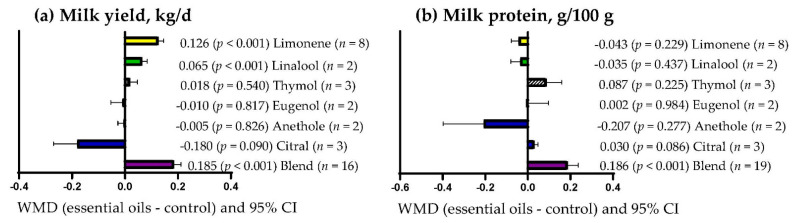
Subgroup analysis (subgroup = primary bioactive compound) of the effect of essential oils supplementation to small ruminants’ diets on their milk yield and composition; WMD = weighted mean differences between essential oil treatments and control.

Figure 6a shows that milk yield increased (*p* < 0.001) only when the primary bioactive metabolites of the EOs offered were mixtures (WMD = 0.185 kg/d), limonene (WMD = 0.126 kg/d) and linalool (WMD = 0.065 kg/d). By contrast, milk yield was not affected when EOs contained other primary bioactive metabolites (*p* > 0.05). Milk protein content increased (*p* < 0.001) when EOs contained mixtures of primary bioactive metabolites (WMD = 0.186 kg/d; Figure 6b) but was not affected when EOs contained other types of bioactive metabolites (*p* > 0.05).

## 4. Discussion

### 4.1. Dry Matter Intake and Digestibility

Supplementation with EOs increased DMI. Similar responses were previously reported by Orzuna-Orzuna et al. [25] in a meta-analysis with beef cattle supplemented with EOs. It has been reported that EOs can improve the taste and palatability of livestock foods [14], which could result in increased DMI. Furthermore, in small ruminants, various EOs have been shown to increase the relative abundance of fungi and ruminal bacteria (*R. flavefaciens*, *R. albus*, and *F. succinogenes*) that are related to fiber degradation in the rumen [41,42]. This could result in a higher rate of feed particle passage in the rumen and higher DMI. Consequently, similar effects of EO consumption in the present meta-analysis would partly explain the observed increase in DMI.

According to Clouard and Val-Laillet [43], the first stimulus perceived by animals when exposed to feed is its aroma. Therefore, EOs should be carefully dosed because some of them have primary bioactive compounds with strong aroma [14], which could limit DMI in ruminants. In addition, a meta-analysis conducted by Orzuna-Orzuna et al. [25] showed that, in beef cattle, the effects of dietary inclusion of EOs on DMI depend on the dose and experimental period used. Nevertheless, in the present meta-analysis, the heterogeneity test for DMI was not significant. This suggests that EOs could be used to stimulate DMI in small ruminants independently of the primary bioactive metabolite, dose, and supplementation period used.

It has been reported that there is a close relationship between the relative abundance of some ruminal microorganisms and the digestibility of dietary nutrients [44]. Thus, one of the objectives of dietary inclusion of EOs is to increase the relative abundance of ruminal microbial populations that utilize efficient fermentation pathways [45], which could improve the efficiency of nutrient utilization. In the present study, dietary supplementation of EOs increased the relative abundance of *R. flavefaciens*, which would explain the increase in DMD, NDFD, and ADFD. Zhou et al. [41] and Kim et al. [46] observed that, in sheep and cattle, dietary supplementation of EOs increased the presence of rumen fungi. These produce high levels of hemicellulases and cellulases [46] and have the ability to penetrate the cell wall to enhance cellulose degradation [47]. Likewise, Zhang et al. [48] reported a higher ruminal concentration of cellulase, lipase, and β-glucosidase in the ruminal fluid of beef cattle supplemented with EOs. In vitro studies [49,50] have shown that EOs increase the relative abundance of *Succinivibrio* bacteria, which have a positive correlation with DMD, NDFD, and ADFD in dairy cows [51]. Furthermore, Cobellis et al [52] reported that, in lambs, dietary supplementation of plants high in EOs decreases the ruminal abundance of *Prevotella* bacteria, which are negatively correlated with CPD in cattle [51]. In the present meta-analysis, similar effects of EO consumption would partially explain the increases observed for DMD, OMD, NDFD, ADFD, and CPD.

### 4.2. Growth Performance and Carcass Characteristics

In the present meta-analysis, dietary inclusion of EOs increased DMI, DMD, OMD, CPD, NDFD, and ADFD, which would partially explain the increase and decrease in ADG and FCR, respectively. In vitro studies [49,50] have reported that EOs increase the relative abundance of bacterial families (*Lachnospiraceae*, *Rikenellaceae*, and *Christensenellaceae*) that are positively and negatively correlated with ADG and FCR, respectively [44,53]. Likewise, some EOs reduce the relative abundance of *Veillonellaceae* bacteria [49], which are negatively correlated with ruminal production of TVFA and ADG in sheep [54,55]. Other additives containing EOs can increase up to 17 and 23% the efficiency of dietary energy utilization for maintenance and weight gain in lambs, respectively [4,16]. Ann et al. [56] and Wu et al. [57] reported that dietary supplementation with EOs in sheep increases the serum concentration of immunoglobulins IgA, IgG, and IgM. This could improve the health status of the animals and consequently increase their productive performance. In addition, previous studies [56,58] have shown that in lambs the dietary inclusion of low doses (50, 80, and 250 mg/kg DM) of EOs increases serum levels of IGF-1 (insulin-like growth factor 1), which is positively correlated with ADG in sheep [59]. For this reason, similar effects of EO consumption in the present study would partially explain the observed improvements in ADG and FCR [59]. 

Dietary supplementation of EOs increases the relative abundance of *Lachnospiraceae* bacteria in bovine rumen fluid [49], which correlates positively with the length of ruminal papillae in sheep [53,55]. Similar effects of EO consumption used in the present meta-analysis could increase ruminal absorption of TVFA and result in higher ADG and lower FCR. Moreover, dietary inclusion of EOs (150 and 300 mg/kg DM) has been reported to increase villus length in the duodenum, jejunum, and ileum of lambs by 37–75% [60]. This could increase the intestinal absorption of amino acids and other nutrients by the animal, which would explain the observed improvements in ADG and FCR.

Dietary inclusion of EOs did not affect HCW, CCW, and BFT but increased HCY and LMA. Similar to our results, a meta-analysis conducted by Orzuna-Orzuna et al. [25] reported that dietary supplementation with EOs increased HCW and LMA in beef cattle, without negatively affecting other carcass characteristics. The increase in HCY observed in the present meta-analysis could be associated with the increase in LMA because there is a positive correlation between these carcass characteristics [61]. Furthermore, according to Laliotis et al. [62], ruminal acetate is the main lipogenic precursor in ruminant adipose tissue. In the present study, the dietary inclusion of EOs did not affect the ruminal acetate concentration, which would explain the absence of significant changes in BFT.

Many of the EOs used in livestock feed are high in terpenoids [5]. It has been reported that terpenoids can promote muscle stem cell differentiation as well as muscle tissue synthesis in mammals [63]. Although there is little information on the mechanisms of action of EOs and their bioactive metabolites on muscle development in ruminants, there is evidence that terpenoids reduce proteolytic degradation in muscle tissue in rats [64] and increase hypertrophy in skeletal muscle cells [65]. Likewise, in beef cattle supplemented with EO mixtures, Monteschio et al. [66] observed increases of 7 and 16% in the diameter and fiber area of *Longissimus dorsi* muscle, respectively. Consequently, similar effects of EO consumption in the present study would partially explain the higher LMA observed.

### 4.3. Ruminal Fermentation and Ruminal Microorganisms

In the present study, dietary supplementation with EOs did not affect rumen pH but reduced rumen NH_3_-N concentration. Like our results, a meta-analysis conducted by Orzuna-Orzuna et al. [25] reported that, in beef cattle, dietary supplementation with EOs reduced rumen NH_3_-N concentration without affecting rumen pH. In the present meta-analysis, the results observed for ruminal pH suggest that ruminal functions of small ruminants were performed under stable conditions because rumen pH serves as an indicator of the internal homeostasis of the ruminal environment [25]. On the other hand, the lower rumen NH_3_-N concentration observed suggests that EOs reduced rumen protein degradation. It has been reported that EOs can reduce the rate of amino acid deamination in the rumen and inhibit the ruminal growth of some hyper ammonia-producing bacteria (*Clostridium sticklandii* and *Peptostreptococcus anaerobius*) [67]. This could reduce rumen ammonia production, which would explain the observed reduction in the ruminal NH_3_-N.

Dietary supplementation with EOs increased the rumen concentration of propionate and reduced the total rumen protozoa population but did not affect the concentration of acetate and butyrate. Like our results, a meta-analysis conducted by Orzuna-Orzuna et al. [25] reported that, in beef cattle, dietary inclusion of EOs reduced the number of protozoa in the rumen and increased the ruminal concentration of propionate, without negatively affecting the other ruminal parameters. The observed increase in ruminal propionate concentration suggests that EOs may increase the availability of energy for growth and production because propionate serves as an energy source for some anabolic functions in ruminants [68]. It has been reported that, under in vitro conditions, dietary inclusion of EOs decreases the relative abundance of *Succiniclasticum* bacteria [49], which are negatively correlated with propionate concentration in rumen fluid [69]. Zhang et al [48] reported that, in beef cattle, dietary supplementation with EOs increased the relative abundance of *Parabacteroides distasonis* and *Bacteroides thetaiotaomicron* bacteria, which increased ruminal propionate concentration. Similar effects of EO consumption in the present meta-analysis would partially explain the higher rumen propionate concentration observed.

The reduction in the number of total protozoa in the rumen could be favorable because an increase in the population of ruminal protozoa increases ruminal protein degradation [70] and CH_4_ emissions [71]. This results in lower utilization efficiency of protein and energy consumed and, consequently, limits the productivity of small ruminants. According to Franzolin and Dehority [72], ruminal pH plays an important role in the survival of rumen protozoa. In the present study, ruminal pH was not affected by the dietary inclusion of EOs. This suggests that the reduction in total protozoa and rumen *Epidinium* might be associated with antimicrobial effects of EOs rather than rumen pH. However, Benchaar et al. [13] mentioned that it is possible that ruminal microorganisms adapt to the effects of EOs when they are used for long periods, which could diminish their positive effects. In the present study, subgroup analysis revealed that total rumen protozoa decreased regardless of the supplementation period used.

In a review article, Cobellis et al. [73] mentioned that, although EOs appear to be effective in reducing the rumen abundance of methanogens, they could also negatively affect the relative abundance of *R. flavefaciens*, *R. albus*, and *F. succinogenes*. However, in the present meta-analysis, EOs increased the abundance of *R. flavefaciens* without affecting the abundance of *R. albus* and *F. succinogenes*. This could be associated with the observed reduction in the total rumen protozoan population, which has been reported to be negatively correlated with the rumen abundance of *R. flavefaciens* in dairy goats [74].

Supplementation of EOs in beef cattle has been reported to increase the relative rumen abundance of the bacterial family *Succinivibrionaceae* [49,50], which has a strong negative correlation with the relative abundance of *Methanobacteriaceae* microorganisms [75]. Similar effects of EO consumption in the present meta-analysis would partially explain the reduction observed for rumen abundance of methanogens. On the other hand, dietary supplementation of EOs reduced enteric CH_4_ emissions in small ruminants. Like our results, a meta-analysis conducted by Belanche et al. [26] reports lower CH_4_ production in dairy cows supplemented with EOs. Wallace et al. [76] demonstrated that, in beef cattle, there is a strong positive correlation between enteric CH_4_ emissions and the relative abundance of rumen methanogens and protozoa. In the present study, EOs reduced the rumen abundance of protozoa and methanogens, which would explain the observed reduction in CH_4_.

### 4.4. Blood Metabolites

In the present meta-analysis, the lower serum urea concentration was observed in response to EOs supplementation. In a previous meta-analysis, Orzuna-Orzuna et al. [25] also reported lower serum urea concentration in beef cattle supplemented with EOs. Additionally, it has been demonstrated that under in vitro conditions, EOs increase the relative abundance of the bacterial family *Lachnospiraceae* [49], which has a negative correlation with serum urea concentration in beef cattle [69]. Similar effects of EO consumption in the present meta-analysis would partially explain the reduction in serum urea concentration. In addition, the lower serum concentration of urea in the present study could be related to the reduction observed in the ruminal concentration of NH_3_-N, because in ruminants these two parameters are positively correlated [77].

According to Ran et al. [78], serum concentrations of glucose, NEFA, and BHB can be used as reliable indicators of energy status in ruminants. In the present meta-analysis, EOs did not affect serum glucose concentration but reduced serum NEFA and BHB concentration. This suggests that dietary supplementation with EOs improves energy balance in small ruminants. Similar responses were previously reported by Orzuna-Orzuna et al. [25] in a meta-analysis with beef cattle supplemented with EOs. The absence of significant changes in serum glucose concentration was not expected because EOs increased the ruminal concentration of propionate, which is the main glucose precursor in ruminants [79]. Additionally, it has been reported that EOs increase the relative abundance of *Lachnospiraceae* and *Bifidobacterium* bacteria [49], which are negatively correlated with serum levels of NEFA [69] and BHB [53] in beef cattle and sheep, respectively. Therefore, similar effects of EO consumption in the present meta-analysis would partially explain the reduction in serum NEFA and BHB concentration.

EOs supplemented in the diet did not affect the serum concentration of albumin, globulin, and total protein, suggesting that supplementation with EOs has no negative effects on protein catabolism and nutritional status of small ruminants [25]. The serum concentration of cholesterol and triglycerides decreased in response to EOs supplementation. There is limited information on the mechanisms of action of EOs on lipid metabolism in small ruminants. However, it has been reported in mice that terpenoids from EOs inhibit hepatic cholesterol biosynthesis and decrease the expression of genes (*Fas*, *Scd1*, and *Acc1*) that are involved in fatty acid synthesis [80]. Similar effects of EO consumption in small ruminants would explain the lower serum cholesterol and triglyceride concentrations in the present study.

In ruminants, excessive accumulation of prooxidant substances such as reactive oxygen species (ROS) can cause oxidative stress [81]. According to Vasta and Luciano [82], it is possible to use EOs as natural antioxidants in the diet of small ruminants because they contain bioactive metabolites (terpenes and terpenoids) with antioxidant properties. In the present study, dietary supplementation with EOs increased TAC. This indicates that EOs reduce ROS in blood serum due to the negative correlation between TAC and ROS in blood [83]. On the other hand, Gessner et al. [84] reported that CAT, SOD, and GPx are antioxidant enzymes that can reduce oxidative stress because they convert ROS into other compounds less harmful to biological macromolecules in the organism. Therefore, in the present meta-analysis, the observed increase in CAT and SOD indicates that EOs reduce oxidative stress in small ruminants.

### 4.5. Meat Quality

Dietary supplementation with EOs did not affect meat pH but reduced CL and ShF. In a previous meta-analysis, Orzuna-Orzuna et al. [25] also found that supplementation with EOs in beef cattle reduced CL and ShF without affecting meat pH. It has been stated that CL can be used as an indicator of water holding capacity (WHC) in ruminant meat [61]. Ablikim et al. [85] reported that CL was negatively correlated (r = −0.894) with WHC in sheep meat. Consequently, the lower CL observed in the present study suggests that supplementation with EOs improves WHC in small ruminant meat. The lower ShF observed in the present meta-analysis suggests that EOs improve tenderness in small ruminant meat [86]. Likewise, the lower ShF observed in response to EOs supplementation could be associated with reduced CL, because in small ruminant meat there is a positive correlation (r = 0.42) between ShF and CL [87].

In the present meta-analysis, EOs reduced b* and MDA in small ruminant meat but did not affect other color parameters (L*, a*) or the chemical composition of meat. Similar responses were previously reported by Orzuna-Orzuna et al. [25] in a meta-analysis with beef cattle supplemented with EOs. Meat color can be used to evaluate the quality of ruminant meat because the color is the first characteristic considered by consumers when choosing fresh meat [88,89]. It has been reported that in lamb meat L* values are associated with the fat content of the meat [90]. Likewise, in beef, Węglarz [91] reported that color parameters (L* and a*) are negatively correlated with meat pH. In the present study, EOs did not affect pH or fat content in small ruminant meat, which would partially explain the absence of changes observed for L* and a*. Furthermore, the observed reduction in b* suggests that EOs improve the quality of fresh small ruminant meat, because consumers generally do not expect to find b* too high in fresh meat [89].

The reduction in MDA in stored meat (on days 1, 3, 6, 9, and 14) suggests that EOs reduce lipid peroxidation of small ruminant meat [92]. According to Pateiro et al. [93], oxidation reactions that occur in meat during storage can cause physicochemical changes and unpleasant odors, which negatively affect meat quality and shelf life. Therefore, the reduction observed for MDA in the present study suggests that EOs can be used as a nutritional strategy to improve the quality and shelf life of small ruminant meat. In addition, previous studies in non-ruminants [94,95] have reported that dietary supplementation with EOs increases antioxidant activity in smooth and skeletal muscle due to increased mRNA for SOD, CAT, and GPx. Likewise, the antioxidant capacity of EOs has been attributed mainly to the terpenoids they contain [5], which after consumption by sheep can be absorbed and deposited in muscle tissues [96]. Similar effects of EO consumption in the present study partially explain the reduction in MDA.

The values observed for the chemical composition of meat suggest that supplementation with EOs does not affect the nutritional value of meat in small ruminants because the nutritional value of meat is related to the content of minerals, fats, and proteins [86]. Thus, the composition of small ruminant meat can be modified by changes in dietary components [61]. In the present study, the dietary inclusion of EOs did not significantly affect the chemical composition of the diets, which would partially explain the observed similarity in the chemical composition of the meat.

According to Dave and Ghaly [97], microbial spoilage of meat can affect its quality and shelf life by negatively affecting pH and appearance, as well as causing off-odors and degradation of structural components. In the present study, supplementation with EOs reduced TVC, ENT, PSY, and MY in meat, suggesting that EOs improve the quality and shelf life of small ruminant meat. Some natural additives have bacteriostatic effects on lamb meat because they reduce its pH [88]. However, in the present meta-analysis, supplementation with EOs did not affect the pH of the meat. It has been shown that terpenoids can be absorbed and deposited in muscle tissues when administered through feed [96]. Terpenoids are known to cause cell lysis and cell death in pathogenic bacteria, as well as inhibit the growth of yeasts and molds [98]. Similar effects of EO consumption in the present study would partially explain the reduction observed for TVC, ENT, PSY, and MY.

### 4.6. Milk Production and Quality

According to Kholif et al. [99], to increase milk yield and FE in small ruminants it is necessary to reduce protein and energy losses during ruminal fermentation, in addition to improving the efficiency of utilization of consumed nutrients. In the present meta-analysis, EOs reduced ruminal NH_3_-N concentration and CH_4_ emissions but increased OMD, CPD, and NDFD. This suggests lower energy and protein loss during ruminal fermentation and higher utilization efficiency of ingested nutrients, which explains the observed increase in milk yield and FE. In addition, under in vitro conditions, EOs increase the relative abundance of microorganisms of the genus *Ruminococcus* [49], which are positively correlated with milk yield in dairy goats [74,100]. It has also been reported that EOs reduce the ruminal abundance of *Clostridium* bacteria in goats [101], which has been negatively correlated with FE in dairy cows [102]. Hence, similar effects of EO consumption in the present study partially explain the observed increases in milk yield and FE [102].

Milk fat content was not affected by supplementation with EOs. In the mammary gland of ruminants, the main precursor for de novo fatty acid synthesis is rumen acetate [103]. Seymour et al. [104] showed that there is a positive correlation (r = 0.31) between milk fat content and ruminal acetate concentration. In the present study, EOs did not affect rumen acetate concentration, which explains the absence of significant effects on milk fat content. On the other hand, higher milk lactose content was observed in response to dietary supplementation with EOs. This could be associated with the observed increase in ruminal propionate concentration because propionate is the main rumen volatile fatty acid required for lactose biosynthesis [99]. Furthermore, in vivo studies with sheep [52] and goats [101] have reported that dietary supplementation with EOs reduces the ruminal concentration of *Prevotella*, which has been negatively correlated with the percentage of lactose in the milk of dairy cows [102]. Zhou et al. [49] observed that in bovine rumen fluid, EOs decrease the relative abundance of *Eubacterium* and methanogens, which is also negatively correlated with the protein content in milk of dairy goats [100]. Consequently, similar effects of EO consumption in the present meta-analysis partially explain the increases observed in lactose and protein content in milk.

SCC can be used as an indicator of udder health and milk quality in ruminants [105]. According to Malik et al. [106], in the milk of healthy ruminants, SCC includes 75–85% immune cells and 15–25% epithelial cells. It has been reported that an increase in SCC is associated with poorer udder health [107] and lower milk quality in ruminants [108]. In the present study, supplementation with EOs reduced SCC, suggesting that EOs could be used to improve udder health and milk quality in small ruminants. In addition, it has been reported that there is a negative correlation between the concentration of antioxidant enzymes and SCC in ruminant milk [106]. In the present meta-analysis, a higher serum concentration of CAT and SOD was observed in small ruminants supplemented with EOs, which would partially explain the observed reduction in SCC.

## 5. Conclusions

The results of the present study indicate that EOs can be used as natural growth promoters in small ruminants and, at the same time, improve feed intake and feed efficiency. Furthermore, dietary supplementation with EOs improves nutrient digestibility, meat quality, and shelf life, as well as milk production and quality. The best result for milk production is obtained with EOs doses lower than 500 mg/kg DM and when the primary bioactive metabolite of the EOs is linalool, limonene, or mixtures of metabolites. Likewise, the best protein content in milk is obtained with supplementation periods longer than 70 days and with the use of EOs that have mixtures of bioactive metabolites.

Dietary supplementation with EOs improves fermentation and reduces environmental impact by increasing ruminal propionate concentration and by reducing methane emissions, ruminal ammonia nitrogen concentration, and the number of total protozoa and methanogens. The best ruminal propionate concentration is obtained when using EOs containing limonene, linalool, or eucalyptol as primary bioactive metabolites. The best result for rumen ammonia nitrogen is obtained with moderate doses (501–1000 mg/kg DM) of EOs and when the primary bioactive metabolite of the EOs is limonene, diallyl disulfide, or mixtures of metabolites. The best results for total protozoa were obtained with supplementation periods longer than 70 days and with the use of EOs having linalool, alpha-pinene, diallyl disulfide, or mixtures of bioactive metabolites. Finally, the results of serum metabolites indicate that EOs improve the antioxidant status in the blood of small ruminants.

## Figures and Tables

**Table 1:** Descriptive statistics of the complete data set for the effect of Eos supplementation on beef cattle diets.

Parameter		Mean		Median		Minimum		Maximum		SD	
Dietary Features	NC	Control	Eos	Control	Eos	Control	Eos	Control	Eos	Control	Eos
Concentrate, g/kg DM	140	479.9	479.9	500.0	500.0	210.0	210.0	790.0	790.0	172.3	172.3
Forage, g/kg DM	140	520.1	520.1	500.0	500.0	100.0	100.0	900.0	900.0	172.3	172.3
DM, g/kg DM	131	863.1	864.1	896.0	896.0	455.0	455.0	973.0	989.0	105.5	106.9
OM, g/kg DM	62	913.1	914.8	912.0	912.0	808.0	808.0	949.0	972.0	24.1	26.2
CP, g/kg DM	106	148.4	147.4	149.5	150.0	80.0	80.0	259.0	253.0	33.1	30.7
EE, g/kg DM	93	29.1	29.4	30.0	30.1	2.6	2.6	63.0	63.0	12.1	12.2
NDF, g/kg DM	102	400.8	400.8	397.3	397.3	118.2	118.2	594.0	594.0	99.6	99.7
ADF, g/kg DM	105	220.8	220.9	225.0	225.0	50.4	50.4	382.3	382.3	62.2	62.2
Starch, g/kg DM	21	225.1	225.1	193.0	193.0	33.0	33.0	405.0	405.0	110.9	110.9
Ca, g/kg DM	79	9.46	9.49	8.0	8.0	1.0	1.0	24.3	24.3	4.93	4.91
P, g/kg DM	79	5.24	5.23	4.2	4.2	1.0	1.0	14.5	14.5	2.83	2.82
ME, Mcal/kg DM	64	2.77	2.74	2.51	2.51	1.48	1.48	4.59	4.54	1.02	1.03
Eos, mg/kg DM	164	-	1452	-	500	-	10	-	40,000	-	3844
Duration, days	162	71		69		14		288		42	

NC = number of comparisons; Eos = essential oils; SD = standard deviation; DM = dry matter; OM = organic matter; CP = crude protein; EE = ether extract; NDF = neutral detergent fiber; ADF = acid detergent fiber; Ca = calcium; P = phosphorus; ME: metabolizable energy. In the same row, means followed by different letters differ significantly by the Tukey test (*p* ≤ 0.05).

**Table 2:** Dry matter intake and nutrient digestibility of small ruminants supplemented with essential oils.

Item	N (NC)				Heterogeneity		Egger Test ^1^
		Control Means (SD)	WMD (95 % CI)	*p*-Value	*p*-Value	I^2^ (%)	*p*-Value
DMI, kg/d	35 (76)	1.146 (0.302)	0.021 (0.013; 0.030)	<0.001	0.115	17.27	0.245
Digestibility, g/kg of DM							
DMD	23 (46)	652.4 (78.8)	14.11 (9.50; 18.72)	<0.001	<0.001	99.24	0.073
OMD	20 (35)	662.5 (81.4)	8.81 (0.08; 17.54)	0.048	<0.001	99.31	0.080
CPD	26 (49)	662.8 (93.1)	12.93 (6.64; 19.21)	<0.001	<0.001	99.64	0.092
EED	9 (18)	631.6 (108.5)	3.13 (−21.32; 27.58	0.802	<0.001	99.86	0.775
NDFD	25 (48)	504.2 (118.6)	13.00 (3.72; 22.28)	0.006	<0.001	99.87	0.116
ADFD	17 (34)	409.5 (123.2)	31.04 (16.51; 45.57)	<0.001	<0.001	99.74	0.066

N: number of studies; NC: number of comparisons; SD: standard deviation; WMD: weighted means differences between control and treatments with essential oils; CI: confidence interval of WMD; *p*-Value to χ2 (Q) test of heterogeneity; I^2^: proportion of total variation of size effect estimates that is due to heterogeneity; ^1^: Egger’s regression asymmetry test; DMI: dry matter intake; DMD: dry matter digestibility; OMD: organic matter digestibility; CPD: crude protein digestibility; EE: ether extract digestibility; NDFD: neutral detergent fiber digestibility; ADFD: acid detergent fiber digestibility.

**Table 3:** Growth performance and carcass characteristics of small ruminants supplemented with essential oils.

Item	N (NC)				Heterogeneity		Egger Test ^1^
		Control Means (SD)	WMD (95 % CI)	*p*-Value	*p*-Value	I^2^ (%)	*p*-Value
ADG, kg/d	21 (51)	0.224 (0.08)	0.008 (0.000; 0.016)	0.037	<0.001	62.34	0.537
FCR, kg/kg	13 (33)	6.54 (3.61)	−0.111 (−0.220; −0.003)	0.045	0.129	22.26	0.075
Carcass characteristics							
HCW, kg	12 (24)	19.68 (5.17)	−0.001 (−0.294; 0.292)	0.996	0.113	28.87	0.906
HCY, %	11 (23)	48.30 (4.51)	0.552 (−0.022; 1.126)	0.049	0.110	27.83	0.306
CCW, kg	8 (17)	17.80 (5.81)	−0.160 (−0.433; 0.113)	0.248	0.184	23.88	0.619
BFT, mm	6 (12)	2.27 (1.11)	−0.033 (−0.152; 0.085)	0.583	0.412	3.39	0.062
LMA, cm^2^	6 (11)	15.07 (4.70)	2.074 (0.674; 3.474)	0.004	<0.001	85.01	0.839

N: number of studies; NC: number of comparisons; SD: standard deviation; WMD: weighted means differences between control and treatments with essential oils; CI: confidence interval of WMD; *p*-Value to χ2 (Q) test of heterogeneity; I^2^: proportion of total variation of size effect estimates that is due to heterogeneity; ^1^: Egger’s regression asymmetry test; ADG: average daily gain; FCR: feed conversion ratio; HCW: hot carcass weight; HCY: hot carcass yield; CCW: cold carcass weight; BFT: backfat thickness; LMA: Longissimus dorsi muscle area.

**Table 4:** Ruminal fermentation and ruminal microorganisms of small ruminants supplemented with essential oils.

Item	N (NC)				Heterogeneity		Egger Test ^1^
		Control Means (SD)	WMD (95 % CI)	*p*-Value	*p*-Value	I^2^ (%)	*p*-Value
pH	31 (78)	6.25 (0.33)	0.00 (−0.037; −0.038)	0.985	<0.001	71.86	0.839
NH_3_-N, mg/dL	29 (69)	19.40 (8.25)	−0.310 (−0.60; −0.02)	0.038	<0.001	62.58	0.241
SCFA, mol/100 mol							
Acetate	30 (73)	5.04 (11.80)	0.165 (−0.71; 1.04)	0.713	<0.001	94.90	0.212
Propionate	30 (73)	21.96 (6.28)	0.726 (0.20; 1.25)	0.006	<0.001	85.88	0.223
Butyrate	30 (73)	11.53 (3.68)	0.050 (−0.24; 0.34)	0.743	<0.001	83.46	0.412
Protozoa, ×10^5^/mL							
Total	14 (34)	7.59 (3.62)	−1.426 (−1.85; −1.00)	<0.001	<0.001	97.91	0.268
*Entodinium*	6 (16)	5.51 (3.14)	−0.008 (−0.05; 0.03)	0.687	<0.001	80.97	0.522
*Diplodidium*	4 (11)	0.49 (0.33)	−0.107 (−0.23; 0.02)	0.094	0.086	40.772	0.177
*Isotrichae*	4 (11)	0.31 (0.08)	0.021 (−0.05; 0.09)	0.574	0.240	21.31	0.074
*Epidinium*	3 (7)	0.85 (0.36)	−0.12 (−0.17; −0.08)	<0.001	0.474	0.00	NA
Microbial population, per mL of ruminal fluid							
Total bacteria, ×10^10^	8 (17)	6.61 (3.24)	0.046 (−0.12; 0.21)	0.579	<0.001	71.65	0.353
*R. flavefaciens*, ×10^8^	6 (11)	9.99 (6.46)	0.43 (0.013; 0.86)	0.043	<0.001	81.33	0.741
*R. albus*, ×10^7^	4 (8)	7.70 (1.55)	0.34 (−0.32; 0.99)	0.311	<0.001	93.94	NA
*F. succinogenes*, ×10^5^	6 (11)	4.99 (2.51)	−0.42 (−0.96; 0.12)	0.129	<0.001	94.18	0.082
Methanogens, ×10^7^	6 (12)	6.319 (2.77)	−0.60 (−0.88; −0.33)	<0.001	<0.001	83.88	0.065
CH_4_, L/d	7 (13)	32.66 (11.71)	−3.93 (−4.68; −3.19)	<0.001	0.352	9.34	0.789

N: number of studies; NC: number of comparisons; SD: standard deviation; WMD: weighted mean differences between control and treatments with essential oils; CI: confidence interval of WMD; *p*-Value to χ2 (Q) test of heterogeneity; I^2^: proportion of total variation of size effect estimates that is due to heterogeneity; ^1^: Egger’s regression asymmetry test; NA: variables with *n* < 10 observations, the test does not apply; NH_3_-N: nitrogen ammonia; SCFA: short-chain fatty acids; CH_4_: enteric methane; R.: Ruminococcus; F.: Fibrobacter.

**Table 5:** Blood metabolites and antioxidant enzymes in blood serum of small ruminants supplemented with essential oils.

Item	N (NC)				Heterogeneity		Egger Test ^1^
		Control Means (SD)	WMD (95 % CI)	*p*-Value	*p*-Value	I^2^ (%)	*p*-Value
Blood metabolites, mg/dL							
Urea	21 (44)	39.07 (15.32)	−0.688 (−1.206; −0.170)	0.009	0.103	21.91	0.978
Glucose	24 (52)	62.52 (18.91)	0.587 (−0.266; 1.440)	0.178	<0.001	79.74	0.306
NEFA, mmol/L	6 (12)	0.361 (0.16)	−0.027 (−0.053; −0.002)	0.034	<0.001	73.76	0.616
BHB, mmol/L	3 (8)	0.446 (0.15)	−0.020 (−0.033; −0.007)	0.003	0.189	29.98	NA
Albumin	17 (32)	4.94 (1.05)	0.029 (−0.003; 0.061)	0.078	0.280	11.70	0.063
Globulin	13 (24)	5.99 (1.81)	0.003 (−0.088; 0.093)	0.953	0.119	29.28	0.253
Protein total	19 (28)	13.31 (2.71)	−0.104 (−0.220; 0.012)	0.080	0.138	48.41	0.305
Cholesterol	20 (45)	114.30 (30.6)	−5.789 (−8.651; −2.926)	<0.001	<0.001	86.83	0.936
Triglycerides	16 (37)	29.90 (10.18)	−2.310 (−3.667; −0.954)	<0.001	<0.001	98.70	0.073
Thyroxine, ng/mL	3 (6)	79.05 (4.33)	7.06 (5.51; 8.61)	<0.001	0.678	0.00	NA
Antioxidant status							
MDA, ng/mL	5 (9)	164.40 (92.50)	−3.88 (−8.48; 0.718)	0.098	0.521	0.00	NA
CAT, ng/mL	4 (7)	1.27 (0.42)	0.204 (0.13; 0.28)	<0.001	0.699	0.00	NA
SOD, ng/mL	6 (12)	1.12 (0.76)	0.037 (0.004; 0.07)	0.028	0.149	31.26	0.642
GPx, nmol/mL	7 (14)	57.20 (39.30)	2.65 (−17.85; 23.15)	0.800	<0.001	99.98	0.346
TAC, U/mL	4 (10)	6.01 (2.45)	0.749 (0.183; 1.31)	0.009	<0.001	85.01	0.811

N: number of studies; NC: number of comparisons; SD: standard deviation; WMD: weighted mean differences between control and treatments with essential oils; CI: confidence interval of WMD; *p*-Value to χ2 (Q) test of heterogeneity; I^2^: proportion of total variation of size effect estimates that is due to heterogeneity; ^1^: Egger’s regression asymmetry test; NA: variables with *n* < 10 observations, the test does not apply; NEFA: non-esterified fatty acids; BHB: beta-hydroxybutyrate; MDA: malondialdehyde; CAT: catalase; SOD: superoxide dismutase; GPx: glutathione peroxidase; TAC: total antioxidant capacity.

**Table 6:** Meat quality of small ruminants supplemented with essential oils.

Item	N (NC)				Heterogeneity		Egger Test ^1^
		Control Means (SD)	WMD (95 % CI)	*p*-Value	*p*-Value	I^2^ (%)	*p*-Value
pH 24 h	15 (26)	5.824 (0.37)	−0.012 (−0.056; 0.033)	0.604	<0.001	77.13	0.080
CL, g/100 g	8 (17)	25.48 (9.02)	−0.617 (−1.174; −0.061)	0.030	0.760	0.00	0.369
ShF, kgf/cm^2^	4 (8)	4.027 (0.20)	−0.171 (−0.337; −0.009)	0.038	0.993	0.00	NA
Meat color							
Lightness (L*)	17 (31)	40.808 (4.69)	−0.207 (−0.505; 0.091)	0.173	0.159	20.61	0.240
Redness (a*)	17 (31)	16.701 (12.29)	0.123 (−0.133; 0.378)	0.347	0.132	22.57	0.359
Yellowness (b*)	15 (29)	6.445 (4.33)	−0.316 (−0.481; −0.151)	<0.001	0.453	0.75	0.860
Lipid oxidation (mg MDA/kg of meat)							
Day 1	12 (24)	0.435 (0.38)	−0.029 (−0.045; −0.014)	<0.001	0.493	0.26	0.069
Day 3	5 (8)	1.591 (1.12)	−0.368 (−0.650; −0.085)	0.011	0.005	65.45	NA
Day 6	9 (20)	2.887 (1.37)	−0.551 (−0.816; −0.286)	<0.001	<0.001	75.02	0..278
Day 9	3 (9)	2.180 (0.76)	−0.189 (−0.337; −0.041)	0.012	0.727	0.00	NA
Day 14	8 (16)	5.888 (2.19)	−1.607 (−2.354; −0.859)	<0.001	<0.001	89.24	0.094
Chemical composition, g/100 g of DM							
Moisture	9 (18)	74.141 (1.48)	0.042 (−0.168; 0.251)	0.696	0.406	4.15	0.288
Protein	9 (18)	25.28 (13.78)	−0.780 (−1.050; −0.509)	0.061	0.198	31.55	0.112
Fat	11 (20)	5.72 (4.70)	0.055 (−0.140; 0.251)	0.578	0.110	30.07	0.223
Ash	8 (16)	1.797 (1.59)	−0.001 (−0.006; 0.004)	0.645	0.702	0.00	0.740
Bacterial counts of raw lamb meat after 7 days of storage, expressed as log CFU/g							
TVC	8 (11)	3.957 (1.98)	−0.605 (−0.857; −0.353)	<0.001	<0.001	68.03	0.480
ENT	6 (9)	1.079 (1.52)	−0.139 (−0.233; −0.045)	0.004	0.805	0.00	NA
PSY	4 (7)	3.084 (0.91)	−0.600 (−0.867; −0.332)	<0.001	0.941	0.00	NA
MY	4 (7)	1.411 (0.45)	−0.275 (−0.537; −0.014)	0.039	0.697	0.00	NA

N: number of studies; NC: number of comparisons; SD: standard deviation; WMD: weighted mean differences between control and treatments with essential oils; CI: confidence interval of WMD; *p*-Value to χ2 (Q) test of heterogeneity; I^2^: proportion of total variation of size effect estimates that is due to heterogeneity; ^1^: Egger’s regression asymmetry test; NA: variables with *n* < 10 observations, the test does not apply; WHC: water holding capacity; CL: cook loss; ShF: shear force; TVC: total viable count of bacteria; PSY: total psychrophilic bacteria; MY: molds and yeast; ENT: Enterobacteriaceae bacteria.

**Table 7:** Milk yield and quality of small ruminants supplemented with essential oils.

Item	N (NC)				Heterogeneity		Egger Test ^1^
		Control Means (SD)	WMD (95 % CI)	*p*-Value	*p*-Value	I^2^ (%)	*p*-Value
Milk yield, kg/d	18 (37)	1.18 (0.76)	0.113 (0.077; 0.148)	<0.001	<0.001	87.35	0.067
FE, kg/kg	10 (21)	0.776 (0.39)	0.039 (0.022; 0.056)	<0.001	0.119	29.56	0.522
Milk composition, g/100 g							
Fat	19 (40)	4.426 (1.33)	−0.003 (−0.099; 0.09)	0.959	<0.001	93.47	0.079
Protein	19 (40)	3.947 (1.15)	0.059 (0.005; 0.113)	0.031	<0.001	91.08	0.424
Lactose	17 (36)	4.811 (0.96)	0.100 (0.048; 0.152)	<0.001	<0.001	86.74	0.269
SCC, ×10^3^ cell/mL	6 (14)	3.081 (1.50)	−0.916 (−1.37; −0.46)	<0.001	<0.001	97.05	0.480
Urea, mg/dL	3 (6)	40.74 (5.46)	−7.73 (−11.77; −3.70)	<0.001	0.043	56.33	NA
pH	3 (6)	6.62 (0.0465)	0.003 (−0.028; 0.034)	0.845	0.989	0.00	NA

N: number of studies; NC: number of comparisons; SD: standard deviation; WMD: weighted mean differences between control and treatments with essential oils; CI: confidence interval of WMD; *p*-Value to χ2 (Q) test of heterogeneity; I^2^: proportion of total variation of size effect estimates that is due to heterogeneity; ^1^: Egger’s regression asymmetry test; NA: variables with *n* < 10 observations, the test does not apply; FE: feed efficiency (kg of milk yield/kg of dry matter intake); SCC: somatic cell count.

**Table 8:** Meta-regression comparing the associations between covariates and measured outcomes.

Parameter	Covariates	QM	Df	*p*-Value	R^2^ (%)
Average daily gain (ADG)	Essential oils dose	0.002	1	0.968	0.0
	Supplementation period	0.824	1	0.364	0.0
	Primary Bioactive Compound	6.56	11	0.834	0.0
Dry matter digestibility (DMD)	Essential oils dose	1.44	1	0.230	0.0
	Supplementation period	3.31	1	0.069	3.23
	Primary bioactive compound	36.01	10	<0.001	17.16
Organic matter digestibility (OMD)	Essential oils dose	1.99	1	0.158	5.86
	Supplementation period	0.258	1	0.612	0.0
	Primary bioactive compound	6.63	8	0.577	0.0
Crude protein digestibility (CPD)	Essential oils dose	0.039	1	0.842	6.61
	Supplementation period	0.479	1	0.489	0.0
	Primary bioactive compound	19.281	11	0.066	0.0
Neutral detergent fiber digestibility (NDFD)	Essential oils dose	3.23	1	0.072	7.15
	Supplementation period	2.35	1	0.125	0.0
	Primary bioactive compound	26.55	11	0.005	7.97
Acid detergent fiber digestibility (ADFD)	Essential oils dose	2.44	1	0.118	4.27
	Supplementation period	0.38	1	0.541	9.29
	Primary bioactive compound	38.50	9	<0.001	62.55
Ruminal pH	Essential oils dose	0.15	1	0.696	0.0
	Supplementation period	8.55	1	0.003	3.52
	Primary bioactive compound	56.31	16	<0.001	56.20
Ammonia nitrogen (NH_3_-N)	Essential oils dose	8.30	1	0.004	16.39
	Supplementation period	2.19	1	0.139	0.0
	Primary bioactive compound	48.30	15	<0.001	40.93
Acetate	Essential oils dose	0.03	1	0.853	0.0
	Supplementation period	3.26	1	0.071	5.78
	Primary bioactive compound	44.27	16	<0.001	7.63
Propionate	Essential oils dose	0.56	1	0.452	2.99
	Supplementation period	1.72	1	0.189	0.0
	Primary bioactive compound	28.69	16	0.026	18.77
Butyrate	Essential oils dose	1.15	1	0.284	3.98
	Supplementation period	0.002	1	0.962	8.65
	Primary bioactive compound	32.71	16	0.008	40.95
Total ruminal protozoa	Essential oils dose	2.43	1	0.119	0.0
	Supplementation period	8.89	1	0.003	7.3
	Primary bioactive compound	31.43	8	<0.001	42.04

QM: coefficient of moderators; QM is considered significant at *p ≤* 0.05; R^2^: the amount of heterogeneity accounted for; Df: degree of freedom.

## Data Availability

The datasets used and analyzed during the current study are available from the corresponding author on reasonable request.

## Appendix A

**Table A1:** Summary of the studies included in the meta-analysis.

Author	Country	Specie	Duration, d	Primary Bioactive Compound	Dose, mg/kg DM
Abd El Tawab et al. [109]	Egypt	Sheep	90	Limonene, thymol	1195, 1272
Abdalla et al. [110]	Brazil	Sheep	28	Blend (*n* = 2)	8333, 15,384
Ahmed et al. [111]	Japan	Sheep	84	Allicin (*n* = 3)	10, 50, 100
An et al. [55]	China	Sheep	60	Blend (*n* = 2)	50, 80
Anasoori et al. [112]	Iran	Sheep	28	Diallyl disulfide (*n* = 2)	500, 750
Anasoori et al. [113]	Iran	Sheep	28	Diallyl disulfide (*n* = 2)	500, 750
Aouadi et al. [114]	Tunisia	Sheep	90	Eucalyptol, Camphor	400, 400
Arteaga-Wences et al. [15]	Mexico	Sheep	56	Blend	129
Bañón et al. [115]	Spain	Sheep	21	Blend	667
Baytok et al. [116]	Turkey	Sheep	56	Carvacrol (*n* = 2)	280, 419
Birick et al. [117]	Turkey	Sheep	70	Carvacrol (*n* = 2), thymol (*n* = 2), blend (*n* = 2)	100 (*n* = 3), 300 (*n* = 3)
Canaes et al. [17]	Brazil	Goats	84	Citral (*n* = 3)	2470, 5007, 7795
Chaves et al. [118]	Canada	Sheep	126	Cinnamaldehyde (*n* = 3)	100, 200, 400
Cobellis et al. [119]	Italy	Sheep	84	Carnosic acid (*n* = 3)	250, 250, 175
Cobellis et al. [52]	Italy	Sheep	84	Carnosic acid (*n* = 3)	250, 250, 175
El-Azrak et al. [19]	Egypt	Goats	45	Blend	750.00
El-Essawy et al. [120]	Egypt	Sheep	120	Anethole, eugenol, thymol	3069, 2920, 2780
El-Essawy et al. [18]	Egypt	Goats	88	Anethole, eugenol, thymol	1706, 1813, 1712
Estrada-Angulo et al. [16]	Mexico	Sheep	87, 100	Blend (*n* = 2)	115, 162
Favaretto et al. [121]	Brazil	Sheep	40	Blend(*n* = 2)	500, 1000
Giannenas et al. [23]	Greece	Sheep	150	Blend(*n* = 3)	50, 100, 150
Güney et al. [57]	Turkey	Sheep	70	Eucalyptol (*n* = 2)	250, 500
Hashem et al. [122]	Egypt	Goats	63	Limonene (*n* = 2)	523, 1051
Hundal et al. [123]	India	Goats	90	Blend	123.00
Jiao et al. [124]	China	Sheep	63	Blend (*n* = 8)	45 (*n* = 4), 79 (*n* = 4)
Kalaitsidis et al. [24]	Greece	Sheep	45	Blend	15
Katheri et al. [125]	Iran	Sheep	48	Blend (*n* = 2)	800, 1600
Khattab et al. [126]	Egypt	Sheep	90	Blend	1232
Kholif et al. [127]	Egypt	Goats	90	Blend (*n* = 3)	1428, 1449, 1428
Kholif et al. [99]	Egypt	Sheep	84	Blend	2475
Kholif et al. [103]	Egypt	Goats	90	Linalool (*n* = 2)	946, 1902
Klevenhusen et al. [128]	Switzerland	Sheep	69	Diallyl disulfide (*n* = 2)	1775, 2000
Kotsampasi et al. [129]	Greece	Sheep	60	Limonene (*n* = 3)	86, 171, 254
Leal et al. [130]	Spain	Sheep	14	Carnosic acid (*n* = 6)	200 (*n* = 2), 400 (*n* = 2), 800 (*n* = 2)
Lei et al. [131]	China	Goats	90	Blend (*n* = 2)	58, 101
Lin et al. [132]	China	Sheep	21	Blend (*n* = 3)	1111, 555, 1111
Ma et al. [133]	China	Sheep	29, 42	Allicin (*n* = 2)	2000 (*n* = 2)
Malekkhani et al. [134]	Iran	Sheep	50	Blend	486
Morsy et al. [135]	Egypt	Goats	90	Blend (*n* = 3)	1428, 1418, 1379
Moura et al. [136]	Brazil	Goats	56	β-caryophyllene (*n* = 3)	500, 1000, 1500
Naseri et al. [42]	Iran	Sheep	56	alpha-pinene	852
Nieto et al. [137]	Spain	Sheep	NR	Thymol (*n* = 2)	1538, 3076
Ortuño et al. [138]	Spain	Sheep	80	Carnosic acid (*n* = 2)	200, 400
Ortuño et al. [139]	Spain	Sheep	80	Blend	400
Ortuño et al. [140]	Spain	Sheep	50	Blend	500
Ortuño et al. [141]	Spain	Sheep	80	Blend (*n* = 2)	200, 400
Ortuño et al. [142]	Spain	Sheep	50	Blend	500
Özdoğan et al. [143]	Turkey	Sheep	56	Blend (*n* = 2)	1000 (*n* = 2)
Panthee et al. [144]	Japan	Sheep	44	Alliin	123
Paraskevakis [145]	Greece	Goats	28	Carvacrol	495
Parvar et al. [146]	Iran	Sheep	90	Blend (*n* = 3)	250, 500, 750
Passetti et al. [147]	Canada	Sheep	100	Blend (*n* = 4)	1100 (*n* = 2), 125 (*n* = 2)
Patindra et al. [148]	Thailand	Goats	42	Eugenol	290
Patra et al. [149]	Germany	Sheep	28	Menthol (*n* = 2)	64, 126
Ranucci et al. [150]	Italy	Sheep	30	Blend	2000
Sahraei et al. [151]	Iran	Sheep	84	Carnosic acid (*n* = 3)	40, 80, 160
Selmi et al. [152]	Tunisia	Sheep	84	Blend (*n* = 2)	150, 300
Serrano et al. [153]	Spain	Sheep	80	Carnosic acid (*n* = 2)	600 (*n* = 2)
Shaaban et al. [20]	Egypt	Sheep	288	Limonene, thymol, blend	1466, 1486, 1476
Simitzis et al. [154]	Greece	Sheep	35	Cinnamaldehyde	413
Smeti et al. [155]	Tunisia	Sheep	60	Eucalyptol	600
Smeti et al. [156]	Tunisia	Goats	56	Blend	599
Smeti et al. [21]	Tunisia	Sheep	100	Blend (*n* = 3)	900, 477, 957
Smeti et al. [22]	Tunisia	Goats	67	alpha-pinene (*n* = 2)	3000, 6000
Soltan et al. [157]	Egypt	Goats	63	Limonene (*n* = 2)	523, 1051
Soltan et al. [77]	Brazil	Sheep	111	Blend (*n* = 2)	200, 400
Ünlü et al. [158]	Turkey	Sheep	56	Blend, capsaicin	300, 300
Wu et al. [56]	China	Sheep	72	Carvacrol (*n* = 2)	2750, 5500
Yanza et al. [159]	Poland	Sheep	48, 30	Rosmarinic acid (*n* = 2)	3920 (*n* = 2)
Yesilbag et al. [160]	Turkey	Goats	60	alpha-pinene (*n* = 3)	400, 800, 2000
Zhang et al. [48]	China	Sheep	24	Carvacrol (*n* = 3)	10,000, 20,000, 40,000
Zhou et al. [41]	China	Sheep	36	Blend (*n* = 2)	52, 91
Zhu et al. [161]	China	Goats	60	Blend	1481
Zhu et al. [162]	China	Goats	30	Blend (*n* = 3)	570, 1140, 1710

DM: dry matter; d: days; *n*: number of treatments.

**Table A2:** Meta-regression comparing the associations between covariates and measured outcomes.

Parameter	Covariates	QM	df	*p*-Value	R^2^ (%)
Meat pH	Essential oils dose	0.80	1	0.370	0.0
	Supplementation period	11.11	1	0.065	0.0
	Primary bioactive compound	97.07	7	<0.001	100
Glucose	Essential oils dose	0.44	1	0.508	1.32
	Supplementation period	0.92	1	0.336	4.64
	Primary bioactive compound	20.54	9	0.015	30.28
Cholesterol	Essential oils dose	1.71	1	0.191	0.0
	Supplementation period	2.31	1	0.128	5.68
	Primary bioactive compound	14.79	10	0.140	0.0
Triglycerides	Essential oils dose	14.64	1	<0.001	2.65
	Supplementation period	1.078	1	0.299	0.0
	Primary bioactive compound	327.36	11	<0.001	80.30
Milk yield	Essential oils dose	22.22	1	<0.001	28.20
	Supplementation period	2.61	1	0.106	0.00
	Primary bioactive compound	38.58	9	<0.001	47.17
Milk fat	Essential oils dose	0.03	1	0.863	0.00
	Supplementation period	5.55	1	0.068	0.00
	Primary bioactive compound	13.05	1	0.071	25.18
Milk protein	Essential oils dose	0.078	1	0.780	0.00
	Supplementation period	6.40	1	0.011	10.41
	Primary bioactive compound	26.17	7	<0.001	32.38
Milk lactose	Essential oils dose	0.826	1	0.363	0.00
	Supplementation period	7.43	1	0.106	2.05
	Primary bioactive compound	13.29	7	0.065	0.00

QM: coefficient of moderators; QM is considered significant at *p ≤* 0.05; R^2^: the amount of heterogeneity accounted for; df: degree of freedom.
